# *Schistosoma mansoni Sm*KI-1 or Its C-Terminal Fragment Induces Partial Protection Against *S. mansoni* Infection in Mice

**DOI:** 10.3389/fimmu.2018.01762

**Published:** 2018-07-30

**Authors:** Suellen B. Morais, Barbara C. Figueiredo, Natan R. G. Assis, Jane Homan, Fábio S. Mambelli, Rodrigo M. Bicalho, Cláudia Souza, Vicente P. Martins, Carina S. Pinheiro, Sergio C. Oliveira

**Affiliations:** ^1^Departamento de Bioquímica e Imunologia do Instituto de Ciências Biológicas, Universidade Federal de Minas Gerais, Belo Horizonte, Brazil; ^2^Instituto Nacional de Ciência e Tecnologia em Doenças Tropicais (INCT-DT), Conselho Nacional de Desenvolvimento Científico e Tecnológico (CNPq), Ministério da Ciência e Tecnologia (MCT), Salvador, Brazil; ^3^Departamento de Bioquímica e Biofísica do Instituto de Ciências da Saúde, Universidade Federal da Bahia, Salvador, Brazil; ^4^ioGenetics LLC, Madison, WI, United States; ^5^Departamento de Biologia Celular do Instituto de Ciências Biológicas, Universidade de Brasília, Brasília, Brazil; ^6^Departamento de Biointeração do Instituto de Ciências da Saúde, Universidade Federal da Bahia, Salvador, Brazil

**Keywords:** *Schistosoma mansoni*, vaccine, *Sm*KI-1, recombinant protein, epitopes

## Abstract

Current schistosomiasis control strategies are mainly based on chemotherapy, but the development of a vaccine against this parasitic disease would contribute to a long-lasting decrease in disease spectrum and transmission. When it comes to vaccine candidates, several genes encoding *Schistosoma mansoni* proteins expressed at the mammalian host–parasite interface have been tested. Among the most promising molecules are the proteins present on the tegument and digestive tract of the parasite. In this study, we evaluate the potential of *Sm*KI-1, the first Kunitz-type protease inhibitor functionally characterized in *S. mansoni*, as a vaccine candidate. Bioinformatic analysis points to the C-terminal fragment as the main region of the molecule responsible for the development of a potential protective immune response induced by *Sm*KI-1. Therefore, for the vaccine formulations, we produced the recombinant (r) *Sm*KI-1 and two different fragments, its Kunitz (KI) domain and its C-terminal tail. First, we demonstrate that mice immunized with recombinant SmKI-1 (r*Sm*KI-1) or its fragments, formulated with Freund’s adjuvant, induced the production of IgG-specific antibodies. Further, all vaccine formulations tested here also induced a Th1-type of immune response, as suggested by the production of IFN-γ and TNF-α by protein-stimulated cultured splenocytes. However, the protective effect conferred by vaccination was only observed in groups which received rSmKI-1 or C-terminal domain vaccines. Mice administered with rSmKI-1 demonstrated reduction of 47% in worm burden, 36% in egg number in mouse livers, and 33% in area of liver granulomas. Additionally, mice injected with C-terminal domain showed reduction of 28% in worm burden, 38% in egg number in liver, and 25% in area of liver granulomas. In contrast, KI domain immunization was unable to reduce worm burden and ameliorate liver pathology after challenge infection. Taken together, our data demonstrated that *Sm*KI-1 is a potential candidate for use in a vaccine to control schistosomiasis, and its C-terminal tail seems to be the main region of the molecule responsible for protection conferred by this antigen.

## Introduction

Schistosomiasis is a disease caused by trematode worms of the genus *Schistosoma* that affects about 207 million people in 76 countries worldwide (1, 2). The main treatment of schistosomiasis is currently the use of anthelmintic drugs, being praziquantel (PZQ) the most widely drug used in chemotherapy. The treatment, although effective, is facing some limitations because the chemotherapy is active only against adult forms of the parasite (not acting on the larval form) and mass treatment does not prevent reinfection (3–5). The lack of new therapeutic drugs and preventive measures, as well as the high disease burden caused by the reinfection are justifications for developing a vaccine against schistosomiasis (4).

Many efforts have been accomplished for the development of an effective vaccine against schistosomiasis (4, 6). Most of the important vaccine targets described up to date are proteins located at the parasite/host interface, since they are commonly associated with mechanisms of escape from the host immune system or other adaptation to parasitism (7) and the two major interfaces are the outer tegument and the gastrodermis (6, 8).

In order to characterize new targets for vaccine development, we decided to perform a pre-clinical study using the recombinant protein *Sm*KI-1. This protein is a Kunitz-type protease inhibitor (KI) recently characterized as an inhibitor for some serine proteases, with possible important functions in anti-coagulation processes (9) and in the parasite immune evasion, through the blockage of neutrophil recruitment (10). In this study, our main goal is to evaluate the vaccine potential of recombinant (r)*Sm*KI-1, and its two fragments: KI domain and C-terminal region, since bioinformatic analysis predicts relevant epitope differences between the KI domain and C-terminal tail. Aiming to test such prediction, we performed vaccination experiments in mice with the whole recombinant protein and its fragments and evaluated the production of specific antibodies, cytokines, and the protection efficacy against schistosomiasis. Our data revealed that vaccination with whole recombinant SmKI-1 (rSmKI-1) and its C-terminal tail, but not its KI domain, induced reduction in worm burden and liver pathology in immunized mice.

## Materials and Methods

### Ethics Statement

All experiments involving animals were conducted in accordance with the Brazilian Federal Law number 11,794, which regulates the scientific use of animals in Brazil, the Institutional Animal Care and Use Committees guidelines and the Animal Welfare Act and Regulations guidelines established by the American Veterinary Medical Association Panel on Euthanasia. Animals were fed, housed, and handled in strict agreement with these recommendations. All protocols were approved by the Committee for Ethics in Animal Experimentation (CETEA) at Universidade Federal de Minas Gerais (UFMG) under permit #185/2017.

### Mice and Parasites

Female C57BL/6 mice aged 6–8 weeks were purchased from the UFMG animal facility. The number of mice used in each individual experiment was calculated taking into account a significance of 0.1%, a test power of 95%, and differences and SDs based on previous literature (11, 12). The number of mice demanded to calculate vaccine efficacy (*n* = 10), or cytokine production (*n* = 5), or antibody titers (*n* = 10), or liver analyses (*n* = 8) were approved by the CETEA at Universidade Federal de Minas Gerais (UFMG) under permit #185/2017. *Schistosoma mansoni* (LE strain) cercariae were routinely maintained in *Biomphalaria glabrata* snails at “Centro de Pesquisa René Rachou Fiocruz (CPqRR)” and prepared by exposing infected snails to light for 2 h to induce shedding of parasites. Cercariae numbers and viability were determined, prior to infection, using a light microscope. Schistosomula were obtained after separation from the tails by centrifugation using a 57% Percoll (Pharmacia, Uppsala, Sweden) solution. Parasites were cultured for at least 7 days *in vitro* as previously described (13).

### Chemicals

All reagents were purchased from Sigma-Aldrich, Co. (St. Louis, MO, USA) unless otherwise specified.

### Accession Number

*Sm*KI-1 (Smp_147730) CCD77156.1.

### Determination of Potential Epitopes for *Sm*KI-1 Antigen

MHC binding was predicted using neural network (NN) ensembles trained to the LN ic50 (14) for over 200,000 binding reactions using the neural platform of JMP^®^ (SAS Institute). This is an updated version of a prediction system described previously (15). Predicted MHC binding affinity was computed for each sequential 9-mer and 15-mer peptide in the protein for 37 MHC-I and 28 MHC-II alleles, including 16 DRB alleles. Binding affinities to inbred murine alleles were similarly determined. To estimate population behavior comprising multiple alleles with varying affinities for any peptide, the LN ic50 binding data estimates were transformed and standardized to a zero mean unit variance within each protein using a Johnson Sb distribution (16). To compute a permuted average across human alleles, the highest predicted binding affinity at each peptide position was determined for every possible haplotype pairing and averaged. B cell linear epitopes were predicted as previously described (17) based on NN predictions trained and cross-validated on the output of a large random peptide set submitted to BepiPred 1.0 (18). The NN provides a structurally based predictor of the probability that an amino acid (within a window ± 4 amino acids) is on the outside of a protein and a likely contact point for a binding antibody based on the biophysical properties of each peptide. The probability of cleavage of each protein by human cathepsin B, L, or S was determined. Both binding affinity and cleavage predictions were accomplished using previously described methods by NN predictors based on principal component analysis of amino acid physical properties (17). T cell exposed motif patterns were extracted from the complete proteomes and ranked as previously described for each of three amino acid recognition patterns which engage T cell receptors (15, 19). Frequencies of motif occurrence were determined with respect to the human immunoglobulinome, based on a data set of over 40 million immunoglobulin variable regions, the human proteome, and the gastrointestinal microbiome (20). To identify potentially suppressive motifs, very common T cell exposed motifs with high binding affinity, or the absence thereof, were identified.

### Synthesis of Recombinant Proteins

The recombinant protein r*Sm*KI-1 and its fragments, Kunitz (KI) domain (N-terminal Arg^22^–Thr^82^) and C-terminal tail (C-terminal Gli^79^–Glu^146^), were produced as previously described (10). Briefly, plasmids containing the sequence for r*Sm*KI-1 or KI-domain or C-terminal tail from SmKI-1 were transformed into *E. coli* Rosetta™ (Merck KGaA, Darmstadt, Germany) competent cells. Cells transformed were cultured in selective medium and gene expression was induced by 1 mM isopropylthiogalactoside (IPTG). After induction, the bacterial cells were harvested and recombinant proteins were recovered as inclusion bodies and solubilized. Each protein was purified by affinity chromatography on a Ni-Sepharose column (Hitrap chelating 5 mL) using an AKTA prime Plus chromatography system (GE Healthcare, São Paulo, Brazil) according to the manufacturer’s protocol. Fractions containing proteins used in this study were determined through SDS/PAGE-20% and then, dialyzed against PBS pH 7.0. The recombinant proteins were quantified using the BCA kit (Pierce, Waltham, MA, USA). To evaluate the amount of endotoxin present, the samples were submitted to Limulus Amebocyte Lysate QCL-1000™ (Lonza) assay. Protein samples show less than 1 endotoxin unit (EU)/mg.

### SDS-PAGE and Immunoblotting

Purified r*Sm*KI-1 and its fragments were analyzed on prepared 12% polyacrylamide SDS-PAGE gels and run as previously described (21). Proteins were then transferred to a Hybond-P PVDF membrane (GE Healthcare, Pittsburgh, PA, USA) (22). The membrane was blocked with TBS-T (tris-buffered saline pH 7.5, 0.05% Tween 20) containing 5% dry non-fat milk powder for 16 h at 4°C. The membrane was then incubated with mouse polyclonal antibodies to r*Sm*KI-1, or KI domain, or C-terminal (diluted 1:100) for 1 h at room temperature. After three washes with TBS-T, the membrane was incubated with goat anti-mouse IgG conjugated to horseradish peroxidase (HRP) (1:2,000) for 1 h at room temperature. After three washes, the membranes were developed using Immobilion™ Western HRP subtract (Millipore Corporation, Billerica, MA, USA) according to the manufacturer’s instructions and visualized in Amersham Imager 600 (GE Healthcare).

### Immunolocalization of *Sm*KI-1 in *S. mansoni* Schistosomula

To immunolocalize *Sm*KI-1, 7-day cultured schistosomula were prepared *in vitro* as described (23). A whole-mount protocol was used, comprising of parasites fixed with −20°C pure acetone for 15 min and washed with saline. Then, schistosomula were blocked with 1% bovine serum albumin (BSA) in phosphate buffered saline (PBST pH 7.2, 0.05% Tween-20) for 1 h. The samples were incubated with anti-r*Sm*KI-1 serum diluted 1:25 in blocking buffer for 1 h. Serum from non-immunized mice was used, in the same dilution, as a negative control. The samples were washed with PBST and incubated with an anti-mouse IgG antibody conjugated to FITC (Molecular Probes, Carlsbad, CA, USA) diluted 1:100 in blocking buffer. Finally, samples were washed, mounted, and viewed using a Nikon Eclipse Ti fluorescence microscope from the “Centro de Aquisição e Processamento de Imagens (CAPI-ICB/UFMG).”

### Mice Immunization, Challenge Infection, and Parasite Burden Assessment

Six- to eight-week-old female C57BL/6 mice were divided into four groups of ten mice each. Mice received vaccine formulation subcutaneously, at the nape of the neck. Each vaccine was prepared with 25 µg of proteins (r*Sm*KI-1, KI domain, or C-terminal) or with a similar volume of PBS (Adjuvant Control Group, ACG), on days 7, 22, and 37, as described in Figure S1A in Supplementary Material. All vaccine preparations, including the negative control (ACG), were formulated using Complete Freund’s Adjuvant (CFA) for the first immunization and Incomplete Freund’s Adjuvant (IFA) for the following two immunizations (Sigma-Aldrich, Co., St. Louis, MO, USA). Fifteen days after the final immunization, mice were anesthetized with 5% ketamine, 2% xylazine, and 0.9% NaCl and challenged with 100 cercariae (LE strain) through percutaneous exposure of the abdominal skin for 1 h. Forty-five days after challenge, adult worms were perfused from the portal veins of each animal and counted as described previously (24). The protection level was calculated by comparing the recovered total worm number, total female number, and total male number from each group in relation to the control group. Two independent experiments were performed.

### Measurement of Specific Antibodies

Following immunization, sera were collected from mice in each experimental group at 2 week intervals. The levels of specific anti-r*Sm*KI-1, anti-KI domain, or anti-C-terminal antibodies were measured by indirect ELISA, as previously described (25). Maxisorp 96-well microtiter plates were coated with 5 µg/mL of each protein in carbonate-bicarbonate buffer (pH 9.6) and then blocked for 2 h at room temperature with PBST (phosphate buffer saline, pH 7.2 with 0.05% Tween-20) plus 10% FBS (fetal bovine serum). Diluted serum (1:100) was added to each well, and plates were then incubated at room temperature. For antibody specificity tests, only samples collected at day 45 (last bleed before cercariae challenge) were used. For each serum, 20 dilutions were evaluated (starting at 1:20 and following a twofold serial dilution). Each dilution was added per well, and plates were then incubated at room temperature. The titer of each sample corresponds to the highest dilution factor that still yields a positive reading, which is the optical density (OD) of 0.010. Titers were expressed by their denominators. Plate-bound antibodies were detected using peroxidase-conjugated anti-mouse IgG, IgG1, or IgG2c (Southern Biotechnology, CA, USA) diluted 1:5,000, 1:10,000, and 1:2,000, respectively. Color reaction was induced by adding OPD (o-phenylenediamine) in citrate buffer (pH 5.0) plus H_2_O_2_ to each well. The color reaction was stopped by adding 5% sulfuric acid to each well. The plates were read at 492 nm in an ELISA plate reader (BioRad, Hercules, CA, USA).

### Cytokine Analysis in Cell Supernatants

The cytokine experiments were performed using cultured splenocytes from individual C57BL/6 mice immunized three times with r*Sm*KI-1, KI domain, C-terminal, or PBS (ACG) (*n* = 5/group). Ten days after the final immunization, mice were euthanized and spleens were collected for further analysis. The cytokine analysis protocol is demonstrated in Figure S1B in Supplementary Material. The splenocytes were maintained in culture with medium alone or stimulated with 5 µg/mL of proteins r*Sm*KI-1, KI domain, or C-terminal. For positive controls, cells were stimulated with either concanavalin A (ConA) (5 µg/mL) or lipopolysaccharide (LPS) (1 µg/mL), as previously described (24). Culture supernatants were collected after 24 h to measure IL-4 and IL-5 levels, after 48 h to measure TNF-α levels, and after 72 h to measure IFN-γ and IL-10 levels. Cytokine measurement assays were performed using the DuoSet ELISA kit (R&D Systems, Minneapolis, MN, USA) according to the manufacturer’s instructions.

### Egg Counts

To evaluate the effect of immunization with r*Sm*KI-1 or its fragments on liver pathology, livers from eight mice per group were collected following perfusion for worm recovery. Liver fragments from each animal were weighed and digested with 10% KOH at 37°C. Released eggs were obtained by centrifugation at 900 *g* for 10 min and resuspended in 1 mL of saline. Egg numbers were counted using a light microscope. Quantification was obtained by calculating the number of eggs per gram of liver tissue.

### Histopathological Analysis

Liver samples taken from the central part of the left lateral lobe were fixed with 10% buffered formaldehyde in PBS. Histological sections were performed using microtome at 6 µm and stained on a slide with haematoxylin-eosin (HE). For measurement of granuloma area, a microscope with 10× objective lens was used and images were obtained through a JVC TK-1270/RBG microcamera attached to the microscope. Twenty granulomas, containing a single well-defined egg were randomly selected in each liver section and the granuloma area was measured using the ImageJ software (U.S. National Institutes of Health, Bethesda, MD, USA, http://rsbweb.nih.gov/ij/index.html).

### Statistical Analysis

Cytokine and antibody analysis were performed using two-way ANOVA and Bonferroni adjustments for comparisons between groups. The results from vaccination experiment (worm burden, egg count, and histopathology) were compared by paired Student’s *t-test*. The *p*-values obtained were considered significant if they were <0.05. Statistical analysis was performed using GraphPad Prism 6 (La Jolla, CA, USA).

## Results

### *S. mansoni Sm*KI-1 Epitope Mapping

**Figure 1:**
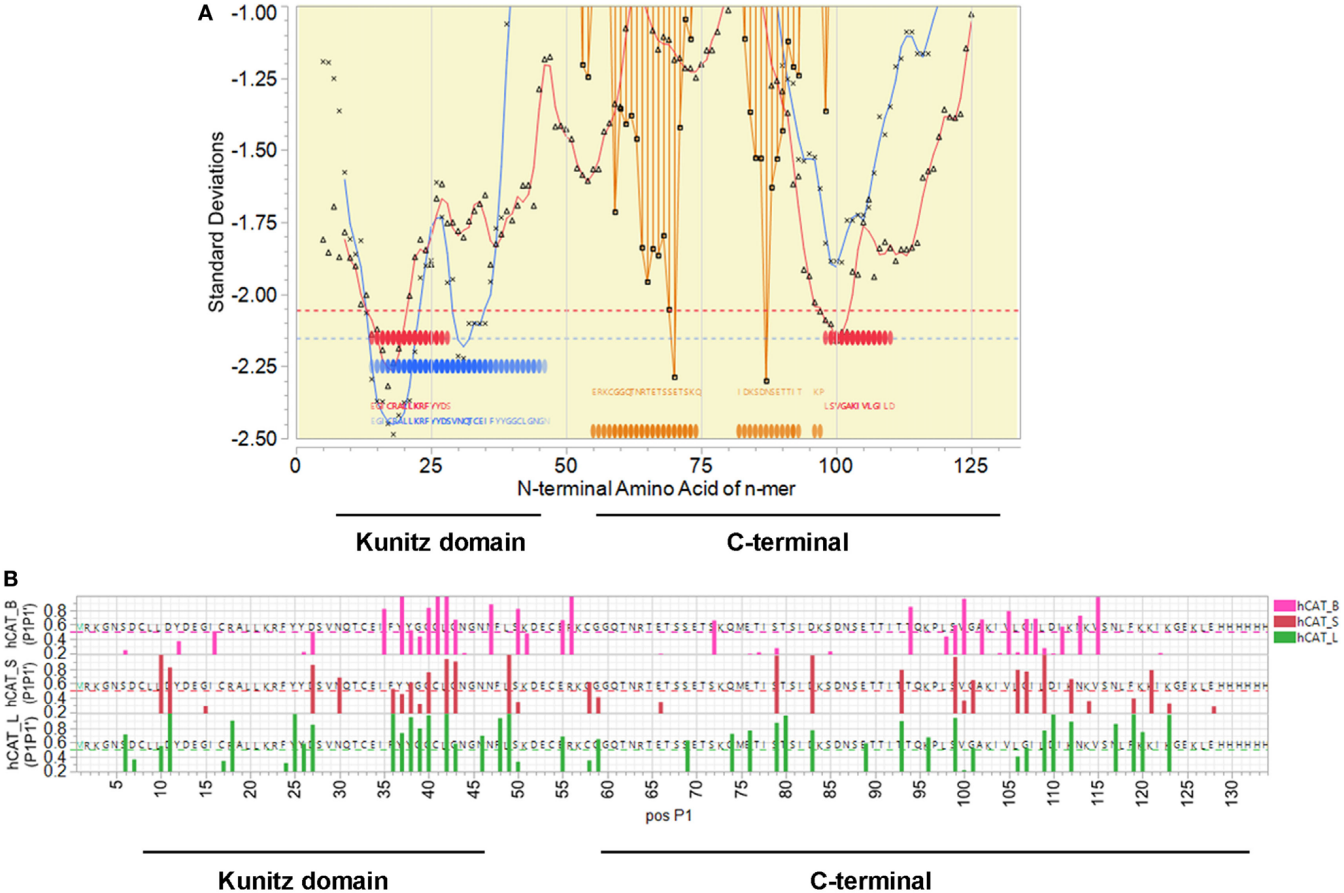
*Sm*KI-1 epitope mapping. **(A)** Representation of the overall epitope mapping of *Sm*KI-1 as presented to a human host. *X* axis indicates sequential peptides with single amino acid displacement. *Y* axis indicates predicted binding affinity in SD units for the protein. Blue lines represent the permuted average of predicted binding of 16 human DRB, in the 15-mer starting at that index position. Red lines indicate the permuted average of predicted binding of 37 human HLA-A and HLA B alleles, in the 9-mer starting at that index position. Blue and red bars across the base line indicate the top 10% of predicted binding peptides. Orange bars indicate probability of a linear B cell epitope starting at that peptide index position. White background indicates signal peptide; yellow the secreted protein. **(B)** Predicted MHC II binding for C57BL/6 H-2-IA^b^ alleles for sequential 15-mer peptides (blue), hashed bars show the peptides predicted to be excised by cathepsin B, L, or S, and probability of B cell linear epitopes (orange). The *Y* axis units for MHC binding are SD units below the mean of the natural log binding affinity (lnIC_50_) for that protein; and the SD of linear B cell epitope probability (inverted).

Mapping of predicted B and T cell epitopes indicates that the most probable B cell epitopes are located in the region of amino acids 89–116 (Figure 1). In this region there are also peptides predicted to have a high affinity binding for many human MHC II DRB alleles, and for murine H2-I-A^b^. Many of the predicted high binding peptides are also predicted to be excised by cathepsins. In contrast the Kunitz domain is lacking in strong B cell epitopes, although it has many peptides with predicted human DRB high affinity binding and some 15-mer peptides predicted to bind to H2-I-A^b^. This indicates that the C-terminal half of the protein is most likely to elicit a strong antibody response.

### *Sm*KI-1 Is Localized on the Surface of the Intestinal Tract of Schistosomula

**Figure 2:**
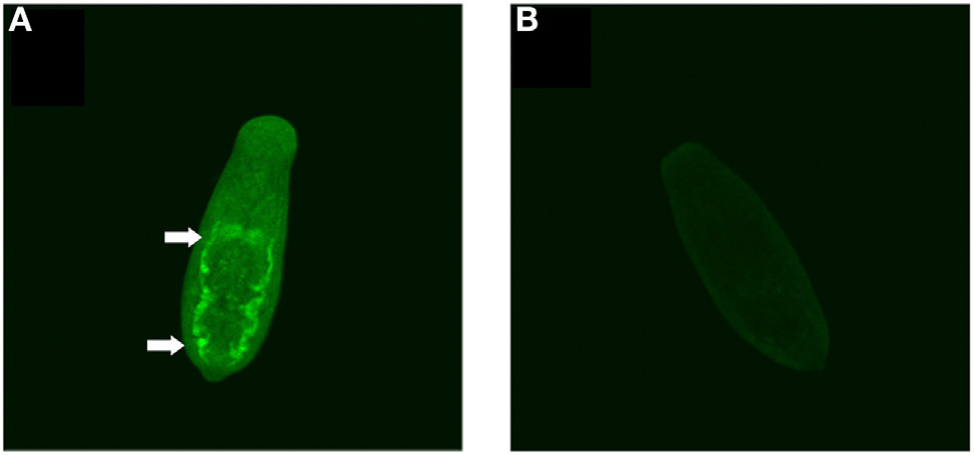
Immunolocalization of *Sm*KI-1 in whole 7-day schistosomula. **(A)** Indirect immunofluorescent labeling of native *Sm*KI-1 in whole fixed schistosomula using mouse polyclonal anti-recombinant SmKI-1 and a secondary anti-mouse conjugated to FITC (green). Arrows indicate clear intestinal staining. **(B)** As a control, serum from naive mice was used.

In a recent study, *Sm*KI-1 was found on the tegument of adult males and females (9). However, there is no information about *Sm*KI-1 localization in schistosomula. Therefore, we stained whole 7-day cultured schistosomula with mouse polyclonal antibodies raised against r*Sm*KI-1 to localize this protein in the larval stage of the parasite. The native *Sm*KI-1 protein (green) was located exclusively in the posterior portion of gut lumen, possibly in the syncytial surface of the gastrodermis of *S. mansoni* schistosomula (Figure 2A). No specific fluorescent signal was detected in the gut or elsewhere when serum from naïve mice (pre-serum) was used in schistosomula from the same source (Figure 2B).

### Vaccination With *Sm*KI-1 and Its Fragments Induces High-Specific IgG Titers

**Figure 3:**
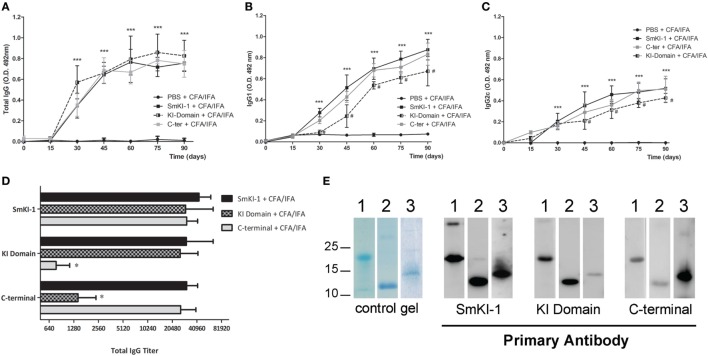
Humoral immune responses to recombinant SmKI-1 (r*Sm*KI-1) or its fragments. Sera from rSmKI-1-vaccinated (black squares), or KI domain-vaccinated (black/gray squares), or from C-terminal-vaccinated (gray squares) and ACG (black circles) mice. Mouse sera (mean ± SD, *n* = 10) were tested by ELISA to evaluate the levels of total IgG antibodies **(A)** or specific IgG1 **(B)** or IgG2c **(C)** antibodies at the indicated time points during the vaccination trial. Asterisks indicate differences detected in protein-immunized group compared to ACG (***p < 0.005) and hashtag indicate differences detected in KI domain-vaccinated group compared to SmKI-1-vaccinated group (^#^*p* < 0.05). **(D)** ELISA plates were coated with rSmKI-1 or its fragments individually and wells were incubated with diluted serum from immunized mice. Anti-IgG from mice were used to detect antibody binding to proteins. The numbers in *X*-axis indicate the dilution factor in a logarithmic scale. Results are presented as the antibody titers (expressed by their denominators) for each group (mean ± SD, *n* = 10). An asterisk indicates differences detected among fragments-vaccinated mice and rSmKI-1-vaccinated group (*p < 0.05). **(E)** Detection by Western blot of rSmKI-1 and its fragments. Lanes contain (1) rSmKI-1, (2) KI domain, and (3) C-terminal proteins. A SDS-PAGE stained with Coomassie is shown as control gel, followed by Western blotting of purified proteins probed with mouse anti-*Sm*KI-1, anti-KI domain, and anti-C-terminal polyclonal antibodies, respectively.

Recombinant *Sm*KI-1 was produced and tested as a vaccine candidate against schistosomiasis. Since bioinformatic analysis pointed to relevant differences between the KI domain and C-terminal tail, each fragment of *Sm*KI-1 was also tested individually. C57BL/6 mice were immunized with 3 doses of each vaccine formulated as 25 µg of each protein (r*Sm*KI-1, KI domain, or C-terminal), or PBS (ACG) in Freund’s adjuvant (CFA/IFA). Sera from 10 animals from each vaccination group were tested by ELISA to evaluate the levels of specific total IgG or IgG subclasses (IgG1 and IgG2c) antibodies to r*Sm*KI-1, KI domain, or C-terminal. From day 30 onward, all groups immunized with the whole protein or its fragments were able to produce higher levels of specific IgG antibodies in comparison to ACG (Figure 3A). All mice vaccinated with r*Sm*KI-1, KI domain and C-terminal, produced significant titers of specific IgG antibodies compared with the control group vaccinated with PBS (ACG) at day 45, pre-challenge with cercariae, and at day 90, before euthanasia as demonstrated in Figure S2 in Supplementary Material. No differences were found among mice which received SmKI-1, KI-domain, or C-terminal antigens. The measurement of IgG isotypes revealed that immunization induced the production of antigen-specific IgG1 (Figure 3B) and IgG2c (Figure 3C) after the second injection. When compared to mice immunized with SmKI-1, KI-domain-immunized animals produced lower levels of both IgG1 and IgG2c. Antibody binding to recombinant proteins was investigated using two different methodologies, ELISA (Figure 3D) and Western blot (Figure 3E). In both experiments, only 45-day serum was used. For ELISA, plates were first coated with r*Sm*KI-1, KI domain, or C-terminal and serum from immunized mice (20 dilutions each) was then added to each well. The data show that antibodies from mice immunized with rSmKI-1, KI domain or C-terminal are able to bind to the whole rSmKI-1 (Figure 3D—top bars). Anti-KI domain antibodies recognize mostly r*Sm*KI-1 and KI domain (Figure 3D—middle bars), while anti-C-terminal antibodies recognize predominantly r*Sm*KI-1 and C-terminal fragment (Figure 3D—bottom bars). For Western blot analysis, purified rSmKI-1 as well as KI domain and C-terminal were resolved by SDS-PAGE (Figure 3E—control gel) and transferred to PVDF membranes. We prepared three replicate membranes. Next, each replicate was incubated with antibodies raised against rSmKI-1, KI domain, or C-terminal. Figure 3E reveals that anti-r*Sm*KI-1 antibodies were able to recognize the whole protein and its fragments, while anti-KI domain and anti-C-terminal antibodies are more specific to its correspondent protein fragment, although they were able to bind rSmKI-1.

### Th1 Cytokine Profile Induced by r*Sm*KI-1, Kunitz Domain, or C-Terminal Fragment Vaccination

**Figure 4:**
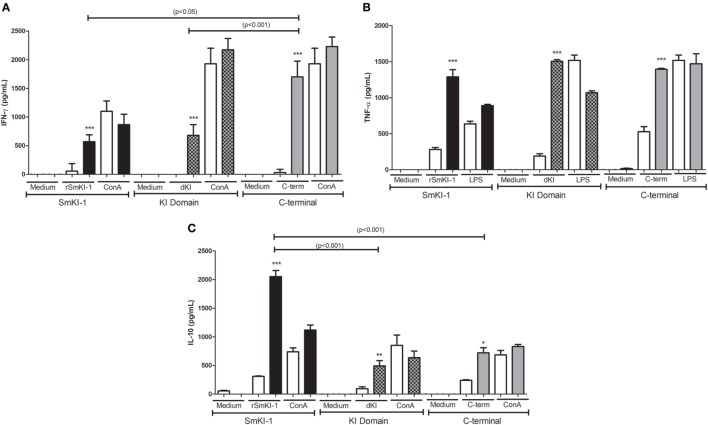
Cytokine profile of splenocytes from recombinant SmKI-1 (r*Sm*KI-1), Kunitz domain, or C-terminal-vaccinated mice. Ten days after the final immunization with r*Sm*KI-1, KI domain, C-terminal, or adjuvant as control, splenocytes from five mice were isolated and assayed for their IFN-γ **(A)**, TNF-α **(B)**, and IL-10 **(C)** production (mean ± SD) in response to stimulation with purified protein (r*Sm*KI-1, KI domain, or C-terminal) or ConA/LPS or medium. Significant differences between data from protein-immunized mice [*Sm*KI-1 + Complete Freund’s Adjuvant (CFA)/Incomplete Freund’s Adjuvant (IFA), KI domain + CFA/IFA, or C-terminal + CFA/IFA] compared to adjuvant-administered (ACG) animals are denoted by *p < 0.05, **p < 0.01, and ***p < 0.005.

Following vaccination with *Sm*KI-1, KI domain, or C-terminal, we measured the production of IFN-γ (Figure 4A), TNF-α (Figure 4B), IL-4, IL-5, and IL-10 (Figure 4C) in supernatants of spleen cells stimulated with purified proteins. In animals immunized with r*Sm*KI-1, we detected higher levels of IFN-γ (764.5 ± 228.0 pg/mL), TNF-α (1288.8 ± 172.8 pg/mL), and IL-10 (2049 ± 243.9 pg/mL) compared to control group. Similarly, KI domain- or C-terminal-immunized mice also presented greater amounts of IFN-γ (613.4 ± 221.0 or 1285.7 ± 339.0 pg/mL, respectively), TNF-α (1506.0 ± 49.0 or 1395.0 ± 309.4 pg/mL), and IL-10 (492.11 ± 161.0 or 721.83 ± 178.3 pg/mL) in comparison to control group. Th2 cytokines, IL-4, and IL-5, were produced in very low levels with no statistically significant differences detected between vaccinated and control groups. ConA or LPS were used as positive controls to confirm that splenocytes were responsive to stimuli (Figure 4). Spleen cells stimulated with medium alone produced no, or negligible cytokine levels (Figure 4). Taken together, these findings suggest that SmKI, KI domain, or C-terminal induced a Th1 type of immune response in vaccinated animals.

### Immunization With r*Sm*KI-1 or C-Terminal Tail, but Not KI Domain Induces Protective Immunity in Mice

**Figure 5:**
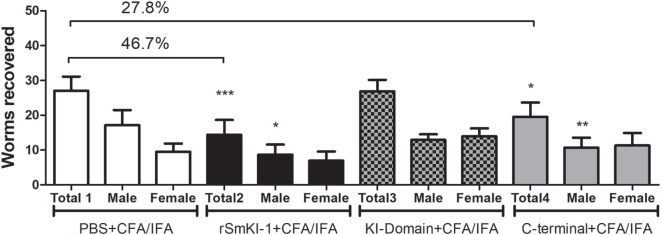
Vaccination with recombinant SmKI-1 (r*Sm*KI-1) or C-terminal reduces the worm burden. Worm burden (mean ± SD) recovered from control mice injected with adjuvant versus mice immunized with rSmKI-1 (black bars) or KI domain (gridded bars) or C-terminal (gray bars) and challenged with *Schistosoma mansoni* cercariae (*n* = 10/group). Graphs represent data from one of two independent vaccine trials. Mice vaccinated with rSmKI-1 or C-terminal showed a 47 or 28% reduction in the adult worm burden, respectively, while mice vaccinated with KI domain showed no difference when compared to ACG. Asterisks indicate statistically significant differences between the protein-vaccinated mice and the control group (***p < 0.005, **p < 0.01, and *p < 0.05).

Two independent vaccination trials with similar results were conducted using C57BL/6 mice and protective immunity was evaluated 45 days after challenge with 100 *S. mansoni* cercariae. The control group received adjuvant only in phosphate buffered saline (ACG). Mice vaccinated with r*Sm*KI-1 showed 46.7% reduction in worm burden (Figure 5—black bars), and C-terminal fragment vaccination reduced in 27.8% of the worm burden (Figure 5—gridded bars), while the KI domain immunization did not induce reduction in recovered parasites (Figure 5—gray bars).

**Figure 6:**
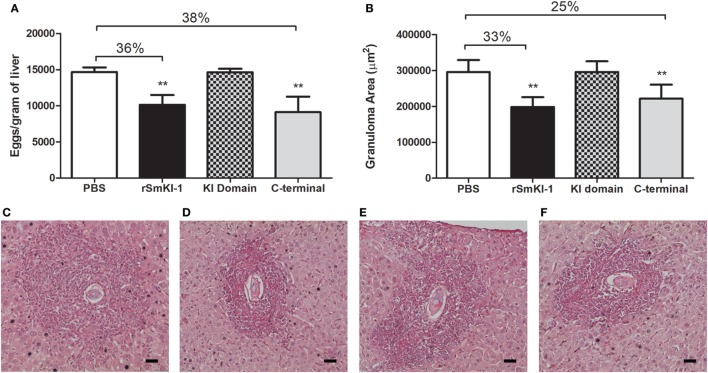
Vaccination recombinant SmKI-1 (r*Sm*KI-1) or C-terminal reduces liver pathology in mice. **(A)** Number of eggs per gram of liver tissue (mean ± SD) of mice (*n* = 8/group). Mice vaccinated with r*Sm*KI-1 or C-terminal fragment showed 38 or 36% reduction in egg numbers, respectively. **(B)** Granuloma area (mean ± SD) in mouse liver sections. Reductions of 33 or 25% are observed after vaccination with r*Sm*KI-1 or C-terminal tail, respectively. Asterisks indicate statistically significant differences between the protein-vaccinated animals compared to the control group (**p < 0.01). Representative images of granulomas detected in a hematoxylin and eosin-stained liver sections from a **(C)** ACG, **(D)** rSmKI-1-vaccinated, **(E)** KI domain-vaccinated, or **(F)** C-terminal-vaccinated animals. The bar represents 0.05 mm.

Regarding the presence of eggs in mouse livers, vaccination with r*Sm*KI-1 reduced the number of eggs per gram of liver tissue by 36% (Figure 6A). A similar reduction, 38% in number of eggs, was also induced in the group vaccinated with the C-terminal. No difference was observed among KI domain-vaccinated mice or ACG. Additionally, analysis of the hepatic tissue demonstrated that *Sm*KI-1 or C-terminal vaccination reduced liver pathology. Histopathological analysis demonstrated that granuloma area was reduced by 33 or 25% in mice which received formulations containing r*Sm*KI-1 or C-terminal, respectively, when compared to mice which received adjuvant only (Figure 6B). For KI domain immunization, no difference to ACG immunization was observed. Figures 6C–F also shows representative images of granulomas from a liver section of ACG, r*Sm*KI-1, KI domain, or C-terminal vaccinated mice.

## Discussion

Recently, our group demonstrated that *Sm*KI-1 is highly expressed in intravascular stages of life cycle, schistosomula, and adult. We also showed that expression of *Sm*KI-1 is essential for parasite survival inside the host, since *Sm*KI-1 siRNA suppression demonstrated a robust impact in schistosome development and survival *in vivo* (10). This protein also presents possible functions in host coagulation processes (9) and in parasite immune evasion (10). Considering that *Sm*KI-1 plays a vital role in parasite survival, we decided to evaluate its potential as vaccine candidate against schistosomiasis.

Bioinformatic analysis of *Sm*KI-1 indicated that the most dominant B cell epitopes are located in the C-terminal fragment of the protein, an observation predictive of the experimental results. Immunolocalization experiments using polyclonal anti-r*Sm*KI-1 indicated that *Sm*KI-1 is mainly present in the intestinal tract in larval stage schistosomula, although it has previously reported to be localized on the tegument in adult worms (9). The intestinal tract is made up of gastrodermis, which consists of a syncytial epithelial layer with many vacuolar compartments that project into the lumen (6, 11). During schistosome blood feeding, the gastrodermis is accessible to host macromolecules such as immunoglobulins (26, 27) which implies direct contact between the digestive epithelia and the host immune system. Gastrodermis proteins were previously tested as vaccines conferring partial protection to mice against schistosomiasis. Sm10.3 antigen and Syntenin partially reduced the worm burden and ameliorated liver pathology (11, 12) and the gut protease SmCB1 reduced significantly worm burden and eggs present in liver and intestine (28). In adult worms, *Sm*KI-1 was localized on the tegument, which is also a major host–parasite interface (7, 8). Numerous tegument antigens have been assessed as vaccine and a previous work from our group achieved a 50% protection testing the antigen Sm29 in murine model (25). Taken together, *Sm*KI-1 epitope mapping and immunolocalization of the protein provide evidence suggesting that *Sm*KI-1 would make a potential vaccine target against schistosomiasis.

In our previous study, we were able to express and purify rSmKI-1, as well as two other protein fragments: KI domain and C-terminal tail (10). Herein, these three proteins were individually used in vaccine formulation associated with Freund’s adjuvant. Mice immunization with these vaccines induced high levels of specific IgG antibodies compared to adjuvant control group. Previous studies associate high levels of IgG isotypes with schistosomula death through antibody-dependent cell-mediated cytotoxicity and the activation of complement (29, 30). The lower levels of both IgG1 and IgG2c produced by KI-domain-immunized mice when compared to SmKI-1-immunized mice are probably related to lower protection against schistosomiasis in the previous group. Antibody production is also involved in long-term protective immunity and related to resistance to schistosomiasis reinfection (31–33). Regarding cytokine profile, supernatants of cultured splenocytes extracted from immunized mice and stimulated with correspondent purified protein yielded high levels of IFN-γ and TNF-α, indicating the development of a Th1-type of immune response. The majority of *S. mansoni* antigens tested as vaccine candidates that conferred partial protection against cercariae challenge induced a Th1-type immune response (24, 25, 34, 35). IFN-γ is a pro-inflammatory cytokine which augments the immune response to schistosome infection by promoting leukocyte activation (24, 36, 37). Macrophage recruitment and activation by IFN-γ is related to worm killing (38) and, together with TNF-α, this cytokine increases the levels of nitric oxide produced by these macrophages in the protective immune response against *S. mansoni* (39). Further, IFN-γ depletion renders mice unable to develop effective defense against schistosome infection (39, 40). Previous studies demonstrate that serine protease inhibitors, when tested as vaccine, are able to induce notable changes in immunological response. The *Toxoplasma gondii* serine protease inhibitor TgPI-1 elicited high titers of IgG antibodies and induced a mixed Th1/Th2 immunological response, with significant production of IFN-γ, when evaluated as a vaccine candidate (41, 42). Another serine protease inhibitor present in the intracellular bacteria *Brucella abortus*, the protein Omp-19, when evaluated as a vaccine candidate, elicited a Th1 immunological response when administrated intraperitoneally, and a mixed Th1/Th17 immunological profile but when administrated orally (43, 44). Finally, *Ancylostoma ceylanicum* Kunitz-type inhibitor (AceKI), also elicited significant titers of IgG in a murine model (45).

Despite high levels of pro-inflammatory cytokines, significant amounts of IL-10 were also produced in experimental-vaccinated groups. IL-10 is an important regulatory cytokine, essential to modulation of the immune response triggered by *S. mansoni* infection (37, 46, 47). This cytokine possibly regulates Th2-type response, reducing inflammation in liver pathology (46). Consistent with that, we detected reduced liver pathology in *Sm*KI-vaccinated mice, group of animals with enhanced IL-10 production. Similarly, the *T. gondii* serine protease inhibitor TgPI-1, when formulated with Alum and CpG-ODN, also induced the production of high levels of IL-10 in a murine model of vaccination (42).

After describing the immune response generated by vaccinated mice, we next investigated whether rSmKI-1 or its fragments could induce protection in a murine model of *S. mansoni* infection. For each protein, two independent experiments were performed. r*Sm*KI-1 conferred a partial protection to infection, resulting in 47% reduction in worm burden. Vaccination experiments using C-terminal region of *Sm*KI-1 resulted in 28% reduction in worm burden, while KI domain trial did not engender protective immunity. Immunization with *Sm*KI-1 or its C-terminal tail also ameliorated liver pathology, since their groups presented 36 and 38% reduction in eggs trapped in liver, respectively. The granuloma area around the eggs was also reduced, 33% upon *Sm*KI-1 vaccination and 25% following C-terminal immunization. Comparatively, the *Boophilus microplus* trypsin inhibitors, containing one or two kunitz proteins, were also able to induce significant protection levels in immunized *Bos indicus*, reaching almost 70% in cattle tick reduction (48). Also, the recombinant serine protease inhibitors TgPI-1, Omp-19, and AceKI were able to induce significant protection levels in murine models of *T. gondii, B. abortus*, and *A. ceylanicum* infections, respectively (42–45).

Previous bioinformatics analysis predicted the C-terminal as a region of unstructured amino acid residues with no similarity with other proteins deposited in the databanks (10). This fragment calls our attention due to epitope prediction which revealed a high probability of recognition by MHC II alleles and significant B cell linear epitopes. Epitope prediction findings seem to corroborate to experimental data, since the best protection results were found in *Sm*KI-1 fragment with higher probability for B and T cell epitopes. The KI domain is highly conserved among species and possesses the protease inhibitor function of *Sm*KI-1 (10). Ranasinghe et al. reported no potential cross-reactivity among SmKI-1 and human TFPI, another Kunitz-type protease inhibitor (49). High identity between host and parasite proteins can be a problem in vaccine development. Despite high antibody titers and the induction of cytokine production, vaccination with KI domain failed to protect mice from schistosome infection, demonstrating no apparent correlation between the anti-KI immune response generated and vaccine protective efficacy.

In summary, the data presented here demonstrate that r*Sm*KI-1 formulated with Freund’s adjuvant generates partial protection against schistosome infection in mice. The protein region correspondent to the C-terminal tail seems to be the region mainly responsible for host immune stimulation, since it seems to have the most important epitopes and generates partial protection against schistosome infection, while the KI domain failed to do so. Taken together, these results support further studies in which *Sm*KI-1, or a fragment thereof, could be optimized and used as a promising vaccine candidate to control schistosomiasis.

## Ethics Statement

All experiments involving animals were conducted in accordance with the Brazilian Federal Law number 11,794, which regulates the scientific use of animals in Brazil, the Institutional Animal Care and Use Committees (IACUC) guidelines and the Animal Welfare Act and Regulations guidelines established by the American Veterinary Medical Association Panel on Euthanasia. Animals were fed, housed, and handled in strict agreement with these recommendations. All protocols were approved by the Committee for Ethics in Animal Experimentation (CETEA) at Universidade Federal de Minas Gerais (UFMG) under permit #185/2017.

## Author Contributions

SM, BF, and SO designed the project and experiments. SM, BF, NA, FM, RB, CS, VM, and CP carried out most of the experiments. SM, BF, and SO wrote the manuscript. SM and BF carried out statistical analysis and prepared figures. JH performed the bioinformatic analysis. SO submitted this paper. All authors reviewed the manuscript.

## Conflict of Interest Statement

JH is an employee and equity holder in ioGenetics LLC. The remaining authors declare that the research was conducted in the absence of any commercial or financial relationships that could be construed as a potential conflict of interest.

## References

[1] McManus DP Loukas A. Current status of vaccines for schistosomiasis. Clin Microbiol Rev (2008) 21(1):225–42. 10.1128/CMR.00046-07 18202444 PMC2223839

[2] Steinmann P Keiser J Bos R Tanner M Utzinger J. Schistosomiasis and water resources development: systematic review, meta-analysis, and estimates of people at risk. Lancet Infect Dis (2006) 6:411–25. 10.1016/S1473-3099(06)70521-7 16790382

[3] Harder A Chemotherapeutic approaches to trematodes (except schistosomes) and cestodes: current level of knowledge and outlook. Parasitol Res (2002) 88(6):587–90. 10.1007/s00436-001-0587-y 12107484

[4] Bergquist NR. Schistosomiasis: from risk assessment to control. Trends Parasitol (2002) 18(7):309–14. 10.1016/S1471-4922(02)02301-2 12379951

[5] Ismail M Botros S Metwally A William S Farghally A Tao LF Resistance to praziquantel: direct evidence from *Schistosoma mansoni* isolated from Egyptian villagers. Am J Trop Med Hyg (1999) 60(6):932–5. 10.4269/ajtmh.1999.60.932 10403323

[6] Figueiredo BC Ricci ND de Assis NR de Morais SB Fonseca CT Oliveira SC. Kicking in the guts: *Schistosoma mansoni* digestive tract proteins are potential candidates for vaccine development. Front Immunol (2015) 6:22. 10.3389/fimmu.2015.00022 25674091 PMC4309203

[7] DeMarco R Verjovski-Almeida S Schistosomes – proteomics studies for potential novel vaccines and drug targets. Drug Discov Today (2009) 14(9–10):472–8. 10.1016/j.drudis.2009.01.011 19429506

[8] Skelly PJ Alan Wilson R. Making sense of the schistosome surface. Adv Parasitol (2006) 63:185–284. 10.1016/S0065-308X(06)63003-0 17134654

[9] Ranasinghe SL Fischer K Gobert GN McManus DP. Functional expression of a novel Kunitz type protease inhibitor from the human blood fluke *Schistosoma mansoni*. Parasit Vectors (2015) 8:408. 10.1186/s13071-015-1022-z 26238343 PMC4524284

[10] Morais SB Figueiredo BC Assis NRG Alvarenga DM de Magalhaes MTQ Ferreira RS *Schistosoma mansoni* SmKI-1 serine protease inhibitor binds to elastase and impairs neutrophil function and inflammation. PLoS Pathog (2018) 14(2):e1006870. 10.1371/journal.ppat.1006870 29425229 PMC5823468

[11] Martins VP Morais SB Pinheiro CS Assis NR Figueiredo BC Ricci ND Sm10.3, a member of the micro-exon gene 4 (MEG-4) family, induces erythrocyte agglutination in vitro and partially protects vaccinated mice against *Schistosoma mansoni* infection. PLoS Negl Trop Dis (2014) 8(3):e2750. 10.1371/journal.pntd.0002750 24651069 PMC3961193

[12] Figueiredo BC Assis NR Morais SB Ricci ND Pinheiro CS Martins VP Schistosome syntenin partially protects vaccinated mice against *Schistosoma mansoni* infection. PLoS Negl Trop Dis (2014) 8(8):e3107. 10.1371/journal.pntd.0003107 25144756 PMC4140676

[13] Da’dara AA Skelly PJ. Gene suppression in schistosomes using RNAi. Methods Mol Biol (2015) 1201:143–64. 10.1007/978-1-4939-1438-8_8 25388112

[14] Vita R Overton JA Greenbaum JA Ponomarenko J Clark JD Cantrell JR The immune epitope database (IEDB) 3.0. Nucleic Acids Res (2015) 43(Database issue):D405–12. 10.1093/nar/gku938 25300482 PMC4384014

[15] Bremel RD Homan EJ. An integrated approach to epitope analysis II: a system for proteomic-scale prediction of immunological characteristics. Immunome Res (2010) 6(1):8. 10.1186/1745-7580-6-8 21044290 PMC2991286

[16] Johnson NL Systems of frequency curves generated by methods of translation. Biometrika (1949) 36(Pt 1–2):149–76. 10.2307/2332539 18132090

[17] Bremel RD Homan EJ. Recognition of higher order patterns in proteins: immunologic kernels. PLoS One (2013) 8(7):e70115. 10.1371/journal.pone.0070115 23922927 PMC3726486

[18] Larsen JE Lund O Nielsen M Improved method for predicting linear B-cell epitopes. Immunome Res (2006) 2:2 10.1186/1745-7580-2-2 16635264 PMC1479323

[19] Bremel RD Homan EJ Frequency patterns of T-cell exposed amino acid motifs in immunoglobulin heavy chain peptides presented by MHCs. Front Immunol (2014) 5:541 10.3389/fimmu.2014.00541 25389426 PMC4211557

[20] Bremel RD Homan J. Extensive T-cell epitope repertoire sharing among human proteome, gastrointestinal microbiome, and pathogenic bacteria: implications for the definition of self. Front Immunol (2015) 6:538. 10.3389/fimmu.2015.00538 26557118 PMC4617169

[21] Laemmli UK Cleavage of structural proteins during the assembly of the head of bacteriophage T4. Nature (1970) 227(5259):680–5. 10.1038/227680a0 5432063

[22] Towbin H Staehelin T Gordon J. Electrophoretic transfer of proteins from polyacrylamide gels to nitrocellulose sheets: procedure and some applications. Proc Natl Acad Sci U S A (1979) 76(9):4350–4. 10.1073/pnas.76.9.4350 388439 PMC411572

[23] Basch PF. Cultivation of *Schistosoma mansoni* in vitro. I. Establishment of cultures from cercariae and development until pairing. J Parasitol (1981) 67(2):179–85. 10.2307/3280633 7241277

[24] Fonseca CT Brito CF Alves JB Oliveira SC. IL-12 enhances protective immunity in mice engendered by immunization with recombinant 14 kDa *Schistosoma mansoni* fatty acid-binding protein through an IFN-gamma and TNF-alpha dependent pathway. Vaccine (2004) 22(3–4):503–10. 10.1016/j.vaccine.2003.07.010 14670333

[25] Cardoso FC Macedo GC Gava E Kitten GT Mati VL de Melo AL *Schistosoma mansoni* tegument protein Sm29 is able to induce a Th1-type of immune response and protection against parasite infection. PLoS Negl Trop Dis (2008) 2(10):e308. 10.1371/journal.pntd.0000308 18827884 PMC2553283

[26] Holtfreter MC Loebermann M Frei E Riebold D Wolff D Hartung G Schistosomula, pre-adults and adults of *Schistosoma mansoni* ingest fluorescence-labelled albumin in vitro and in vivo: implication for a drug-targeting model. Parasitology (2010) 137(11):1645–52. 10.1017/S0031182010000405 20500919

[27] Li XH de Castro-Borges W Parker-Manuel S Vance GM Demarco R Neves LX The schistosome oesophageal gland: initiator of blood processing. PLoS Negl Trop Dis (2013) 7(7):e2337. 10.1371/journal.pntd.0002337 23936568 PMC3723592

[28] El Ridi R Tallima H Selim S Donnelly S Cotton S Gonzales Santana B Cysteine peptidases as schistosomiasis vaccines with inbuilt adjuvanticity. PLoS One (2014) 9(1):e85401. 10.1371/journal.pone.0085401 24465551 PMC3897446

[29] Khalife J Dunne DW Richardson BA Mazza G Thorne KJ Capron A Functional role of human IgG subclasses in eosinophil-mediated killing of schistosomula of *Schistosoma mansoni*. J Immunol (1989) 142(12):4422–7. 2723436

[30] Capron A Bazin H Dessaint JP Capron M. [The role of specific IgE antibodies in the immune adherence of normal macrophages to schistosomes of *Schistosoma mansoni*]. C R Acad Sci Hebd Seances Acad Sci D (1975) 280(7):927–30. 809179

[31] Melo TT Sena IC Araujo N Fonseca CT. Antibodies are involved in the protective immunity induced in mice by *Schistosoma mansoni* schistosomula tegument (Smteg) immunization. Parasite Immunol (2014) 36(2):107–11. 10.1111/pim.12091 24558655

[32] Zhang W Ahmad G Le L Rojo JU Karmakar S Tillery KA Longevity of Sm-p80-specific antibody responses following vaccination with Sm-p80 vaccine in mice and baboons and transplacental transfer of Sm-p80-specific antibodies in a baboon. Parasitol Res (2014) 113(6):2239–50. 10.1007/s00436-014-3879-8 24728521 PMC8592056

[33] Gaze S Driguez P Pearson MS Mendes T Doolan DL Trieu A An immunomics approach to schistosome antigen discovery: antibody signatures of naturally resistant and chronically infected individuals from endemic areas. PLoS Pathog (2014) 10(3):e1004033. 10.1371/journal.ppat.1004033 24675823 PMC3968167

[34] Garcia TC Fonseca CT Pacifico LG Duraes Fdo V Marinho FA Penido ML Peptides containing T cell epitopes, derived from Sm14, but not from paramyosin, induce a Th1 type of immune response, reduction in liver pathology and partial protection against *Schistosoma mansoni* infection in mice. Acta Trop (2008) 106(3):162–7. 10.1016/j.actatropica.2008.03.003 18423420

[35] Farias LP Cardoso FC Miyasato PA Montoya BO Tararam CA Roffato HK *Schistosoma mansoni* stomatin like protein-2 is located in the tegument and induces partial protection against challenge infection. PLoS Negl Trop Dis (2010) 4(2):e597. 10.1371/journal.pntd.0000597 20161725 PMC2817717

[36] Hoffmann KF Cheever AW Wynn TA. IL-10 and the dangers of immune polarization: excessive type 1 and type 2 cytokine responses induce distinct forms of lethal immunopathology in murine schistosomiasis. J Immunol (2000) 164(12):6406–16. 10.4049/jimmunol.164.12.6406 10843696

[37] Araujo JM de Melo TT de Sena IC Alves CC Araujo N Duraes Fdo V *Schistosoma mansoni* schistosomula tegument (Smteg) immunization in absence of adjuvant induce IL-10 production by CD4+ cells and failed to protect mice against challenge infection. Acta Trop (2012) 124(2):140–6. 10.1016/j.actatropica.2012.07.007 22842304

[38] Jankovic D Wynn TA Kullberg MC Hieny S Caspar P James S Optimal vaccination against *Schistosoma mansoni* requires the induction of both B cell- and IFN-gamma-dependent effector mechanisms. J Immunol (1999) 162(1):345–51. 9886405

[39] Pearce EJ MacDonald AS The immunobiology of schistosomiasis. Nat Rev Immunol (2002) 2(7):499–511. 10.1038/nri843 12094224

[40] Smythies LE Coulson PS Wilson RA. Monoclonal antibody to IFN-gamma modifies pulmonary inflammatory responses and abrogates immunity to *Schistosoma mansoni* in mice vaccinated with attenuated cercariae. J Immunol (1992) 149(11):3654–8. 1431135

[41] Sanchez VR Fenoy IM Picchio MS Soto AS Arcon N Goldman A Homologous prime-boost strategy with TgPI-1 improves the immune response and protects highly susceptible mice against chronic *Toxoplasma gondii* infection. Acta Trop (2015) 150:159–65. 10.1016/j.actatropica.2015.07.013 26200784

[42] Cuppari AF Sanchez V Ledesma B Frank FM Goldman A Angel SO *Toxoplasma gondii* protease inhibitor-1 (TgPI-1) is a novel vaccine candidate against toxoplasmosis. Vaccine (2008) 26(39):5040–5. 10.1016/j.vaccine.2008.07.031 18675873

[43] Ibanez AE Coria LM Carabajal MV Delpino MV Risso GS Cobiello PG A bacterial protease inhibitor protects antigens delivered in oral vaccines from digestion while triggering specific mucosal immune responses. J Control Release (2015) 220(Pt A):18–28. 10.1016/j.jconrel.2015.10.011 26456256

[44] Pasquevich KA Ibanez AE Coria LM Garcia Samartino C Estein SM Zwerdling A An oral vaccine based on U-Omp19 induces protection against *B. abortus* mucosal challenge by inducing an adaptive IL-17 immune response in mice. PLoS One (2011) 6(1):e16203. 10.1371/journal.pone.0016203 21264260 PMC3021544

[45] Chu D Bungiro RD Ibanez M Harrison LM Campodonico E Jones BF Molecular characterization of *Ancylostoma ceylanicum* Kunitz-type serine protease inhibitor: evidence for a role in hookworm-associated growth delay. Infect Immun (2004) 72(4):2214–21. 10.1128/IAI.72.4.2214-2221.2004 15039345 PMC375216

[46] Sadler CH Rutitzky LI Stadecker MJ Wilson RA. IL-10 is crucial for the transition from acute to chronic disease state during infection of mice with *Schistosoma mansoni*. Eur J Immunol (2003) 33(4):880–8. 10.1002/eji.200323501 12672053

[47] Teixeira-Carvalho A Martins-Filho OA Peruhype-Magalhaes V Silveira-Lemos D Malaquias LC Oliveira LF Cytokines, chemokine receptors, CD4+CD25HIGH+ T-cells and clinical forms of human schistosomiasis. Acta Trop (2008) 108(2–3):139–49. 10.1016/j.actatropica.2008.04.010 18534548

[48] Andreotti R Cunha RC Soares MA Guerrero FD Leite FP de Leon AA. Protective immunity against tick infestation in cattle vaccinated with recombinant trypsin inhibitor of *Rhipicephalus microplus*. Vaccine (2012) 30(47):6678–85. 10.1016/j.vaccine.2012.08.066 22959980

[49] Ranasinghe SL Duke M Harvie M McManus DP. Kunitz-type protease inhibitor as a vaccine candidate against schistosomiasis mansoni. Int J Infect Dis (2018) 66:26–32. 10.1016/j.ijid.2017.10.024 29128645

