# Supplementary material for: Schistosoma mansoni SmKI-1 or Its C-Terminal Fragment Induces Partial Protection Against S. mansoni Infection in Mice
Source: Front Immunol. 2018 Jul 30;9:1762. doi: 10.3389/fimmu.2018.01762 (PMC6077287; doi:10.3389/fimmu.2018.01762)
Supplement: Supplementary file 1 [file data_sheet_1.PDF]

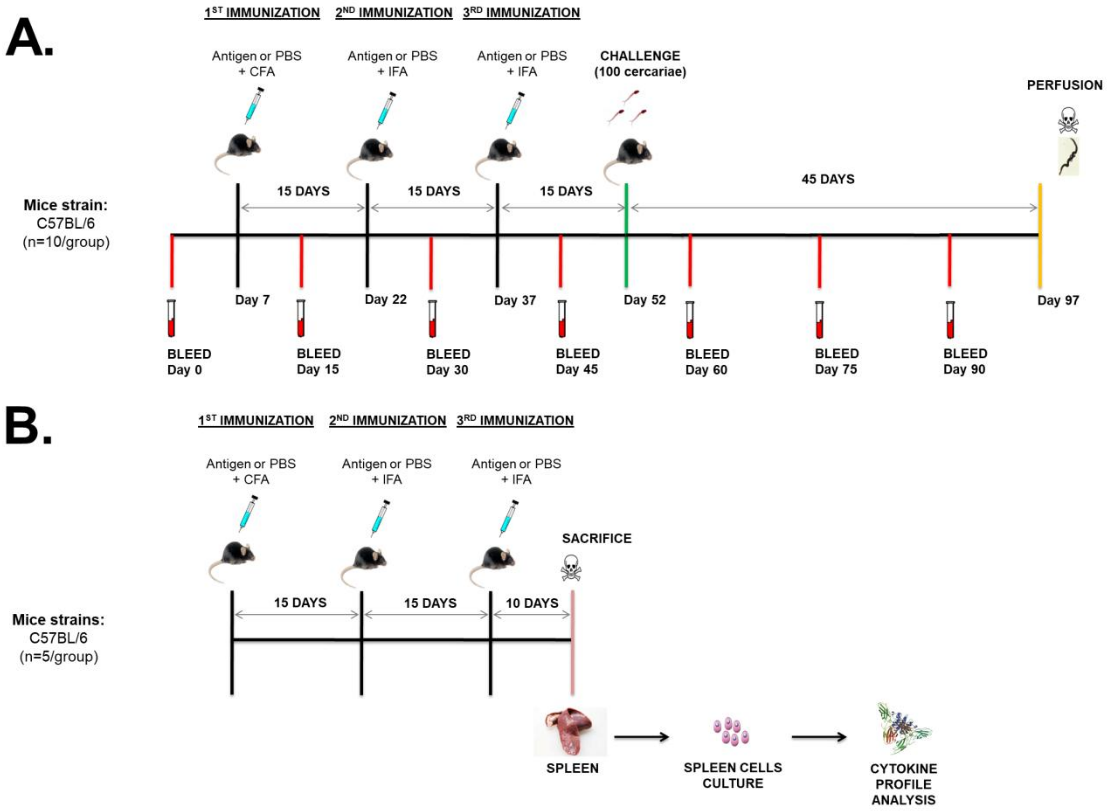

Supplementary Figure 1. Vaccination scheme. (A) Groups of 10 mice each (female C57BL/6 aged 8 weeks) were subcutaneously injected in the nape of the neck with 25µg of recombinant proteins or PBS, for adjuvant control group (ACG), on days 7, 22 and 37. Each protein or ACG was formulated with Complete Freund's Adjuvant (CFA) for the first immunization and Incomplete Freund's Adjuvant (IFA) for the last two immunizations. Fifteen days after the last immunization, on day 52, the mice were challenged through percutaneous exposure of abdominal skin to water containing 100 cercariae for 1 hour. Forty-five days after the challenge, adult worms were perfused from the portal veins of each animal. Two independent experiments were performed to determine vaccine efficacy. (B) Groups of 5 mice each (female C57BL/6 aged 8 weeks) were subcutaneously injected in the nape of the neck with 25µg of recombinant proteins or PBS, for ACG with a 15-day interval from one immunization to another. Each protein or ACG was formulated with Complete Freund's Adjuvant (CFA) for the first immunization and Incomplete Freund's Adjuvant (IFA) for the last two immunizations. Ten days after the last immunization, mice were sacrificed and the spleens were collected for cytokines evaluation. Two independent experiments were performed.
