# Supplementary material for: Schistosoma mansoni SmKI-1 or Its C-Terminal Fragment Induces Partial Protection Against S. mansoni Infection in Mice
Source: Front Immunol. 2018 Jul 30;9:1762. doi: 10.3389/fimmu.2018.01762 (PMC6077287; doi:10.3389/fimmu.2018.01762)
Supplement: Supplementary file 2 [file data_sheet_2.PDF]

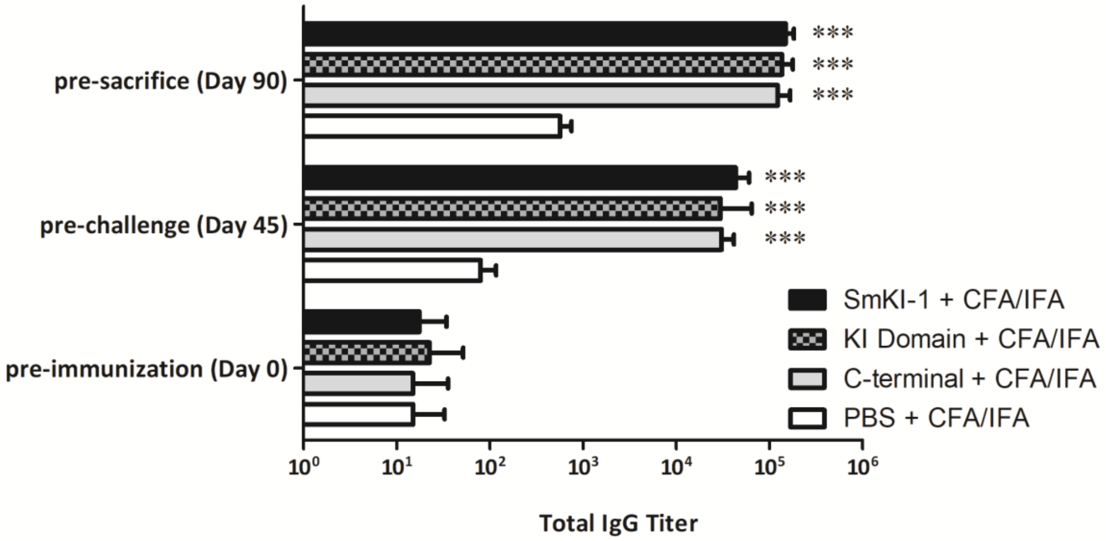

Supplementary Figure 2. Evaluation of antibody titers generated by immunizations r*Sm*KI-1 or its fragments. Sera collected pre-sacrifice (day 90), pre-challenge (day 45) and pre-immunization (day 0) from r*Sm*KI-1-vaccinated (black squares), KI domain-vaccinated (black/gray squares) or from C-terminal-vaccinated (gray squares) and ACG (white squares) mice. Mouse sera (mean ± SD, n = 10) were tested by ELISA to evaluate the antibody titers. ELISA plates were coated with r*Sm*KI-1 and wells were incubated with diluted serum from immunized mice. Anti-IgG from mice were used to detected antibody binding to protein. The numbers in X-axis indicate the dilution factor in a logarithmic scale. Results are presented as the antibody titers (expressed by their denominators) for each group (mean ± SD, n = 10). Asterisks indicate differences detected in protein-immunized group compared to ACG (***$p<0.005$).
